# Clinical outcome of Descemet membrane endothelial keratoplasty (DMEK) with imported donor corneas in eyes of Asian patients; endothelium‐in versus endothelium‐out method

**DOI:** 10.1371/journal.pone.0270037

**Published:** 2022-06-30

**Authors:** Young-ho Jung, Chang Ho Yoon, Mee Kum Kim

**Affiliations:** 1 Department of Ophthalmology, Seoul National University College of Medicine, Jongno-gu, Seoul, Republic of Korea; 2 Department of Ophthalmology, Seoul National University Hospital, Jongno-gu, Seoul, Republic of Korea; 3 Laboratory of Ocular Regenerative Medicine and Immunology, Biomedical Research Institute, Seoul National University Hospital, Jongno-gu, Seoul, Republic of Korea; 4 Transplantation Research Institute, Seoul National University Medical Research Center, Jongno-gu, Seoul, Korea; University of Missouri-Columbia, UNITED STATES

## Abstract

**Objective:**

We investigated whether (1) imported pre-cut tissue is feasible for Descemet membrane endothelial keratoplasty (DMEK) in eyes of Asian patients, (2) the clinical outcome is comparable between the endothelium‐in and endothelium‐out methods, and (3) the corneal edema-induced anterior curvature changes may have an effect on the refractive error.

**Methods:**

The medical records of 32 DMEK patients who underwent either the endothelium-out or endothelium-in method using imported pre-cut grafts with a 3-day pre-cut-to-use time were retrospectively analyzed. Fuchs’ endothelial dystrophy (37.5%) and bullous keratopathy (62.5%) cases were included. The main clinical outcome measures were graft survival, best corrected visual acuity (BCVA), endothelial cell density (ECD), corneal thickness (CT), and complications. Correlation of the anterior curvature changes with refractive error was analyzed in the DMEK with cataract surgery group.

**Results:**

The overall survival rate was 71.9%. Final graft failures were caused by rejection, glaucoma, and infection. Visual acuities improved by 89.3%. BCVA better than 20/40 and 20/20 was found in 75% and 28.6% of patients, respectively. The ECDs at 3 months and 1 year were 1400 and 1083 cells/mm^2^, respectively. The mean survival time, ECD, BCVA, CT, and complication rates were not different between the endothelium-in and endothelium-out methods. A hyperopic shift by +0.42 D was not related to the anterior curvature changes.

**Conclusion:**

Imported pre-cut tissues with a ≤ 3-day pre-cut-to-use time are feasible for DMEK in the treatment of corneal endothelial edema in eyes of Asian patients, and both endothelium-in and endothelium-out methods appear to be comparatively effective. Edema-induced anterior curvature change may not affect the refractive shift.

## Introduction

Since Gerrit Melles first proposed the concept of posterior lamellar keratoplasty in 1998 [1], endothelial keratoplasty has been prevalent in Western countries for both bullous keratopathy (BK) and Fuchs’ endothelial dystrophy (FED) [2, 3]. After the introduction of Descemet membrane endothelial keratoplasty (DMEK) by Gerrit Melles [4–6], faster and better visual recovery and a lower rate of graft rejection were noted, compared to either Descemet stripping automated endothelial keratoplasty (DSAEK) or penetrating keratoplasty [2, 3, 7]. Currently, nearly 850 articles related to DMEK are available on PubMed. Among them, hundreds of articles present the clinical outcomes of DMEK in Western countries. There are presently only about 20 articles that describe the clinical outcomes of DMEK in Asian countries. The low popularity of DMEK in Asia could be related to multiple causes such as the low surgical volume owing to the shortage of donor corneas, the surgically challenging small dimension of the eyes, and the rarity of FED cases in Asia [8–13].

Since various surgical techniques are employed in DMEK to reduce the endothelial cell loss [8, 14–19], standardization of the DMEK procedure is necessary, especially for the challenging cases of Asian eyes. In Korea, there are very few native corneal donors. Therefore, patients who wish to undergo DMEK prefer opting for imported tissues to ensure a timely operation rather than waiting longer for a native tissue. Unlike Western countries, where pre-cut tissue is available within 24–48 hours following the stripping procedure, overseas transport of pre-cut tissues from eye banks in Western countries to Asia takes more than 2 days. It is not fully elucidated whether the overseas transport of pre-cut tissues, with more than a 48-hour period between the pre-cutting and the use time, affects the clinical outcome of DMEK in eyes of Asian patients.

Additionally, the refractive outcome and factors causing shift in the refractive errors have been currently debated in patients with FED following DMEK combined with cataract surgery [20–23]. Various incidents of hyperopic surprise have been reported, and preoperative thicker cornea or postoperative flatter posterior curvature have been suspected to be the underlying risk factors [20, 22]. However, it is still not clear whether edema-induced anterior curvature changes have an effect on the refractive errors in patients with corneal edema.

Overall, three issues need to be addressed: (1) whether imported pre-cut tissues are feasible for use in DMEK in Asia, (2) whether the clinical efficacy of the endothelium‐in delivery method is comparable to that of the endothelium‐out method in DMEK, and (3) the extent of shift in the refractive error following DMEK and whether it is related to the anterior curvature changes. Therefore, this study investigated 1) the clinical efficacy of DMEK in eyes of Asian patients using imported pre-cut tissues with a ≤ 3-day pre-cut-to-use time, 2) the correlation of the anterior curvature changes with refractive shift in DMEK combined with cataract surgery, and 3) whether the clinical efficacy of the endothelium‐in delivery is comparable to that of the endothelium‐out method in DMEK with imported pre-cut tissues. These results will benefit the standardization of DMEK procedures in eyes of Asian patients using imported pre-cut tissues.

## Materials and method

### Subjects

The study adhered to the tenets of the Declaration of Helsinki. Informed consent was waived by the Institutional Review Board because the study was based on the retrospective review of old charts. Under the approval by the Institutional Review Board of Seoul National University Hospital (IRB No. 2108-026-1241), the medical records of DMEK cases performed by one surgeon (M.K.K) at our tertiary referral center between October in 2016 and May 6^th^ in 2022 were retrospectively reviewed.

Patients who had been diagnosed with corneal endothelial edema due to either FED or BK and had undergone DMEK with ≥6 month-follow up were included. The surgical indication was persistent corneal stromal edema with decreased visual acuity. Among the 35 DMEK cases in the above-mentioned period, three cases were excluded from the analysis. Two were excluded owing to loss in follow-up (< 6 months), and the remaining was excluded because the patient had previously undergone whole corneal transplantation prior to DMEK with a high risk of rejection. Of the total 32 cases, one-third had grade 4 FED with corneal stromal edema (n = 12, 37.5%), and two-third had BK (n = 20, 62.5%) [24, 25]. Eyes with previous posterior capsular openings or those that had undergone a previous vitrectomy were also included. (n = 4, 12.5%). DMEK combined with cataract surgery was performed in 18 cases, concomitantly (2 cases) or sequentially (16 cases). Pupilloplasty was performed sequentially in 3 cases before DMEK. All the patients’ demographics are presented in Table 1. Although the indications of the patients were heterogenous (either FED or bullous keratopathy) in each group, there was no statistical difference in the distribution of the previous diagnosis between these two groups.

**Table 1:** Preoperative demographics in recipients and donors.

Characteristics	Total (n = 32)	Endo-out (n = 17)	Endo-in (n = 15)	P value
**Recipients**				
Age (years)	64.5±11.1	62.9±10.8	66.3±11.5	0.396
Sex (M:F)	13: 19	6: 11	7: 8	0.720
Preoperative BCVA (logMAR)	0.92±0.4	0.84±0.4	1.01±0.4	0.630
Surgical indication (Fuchs’ vs BK)	12: 20	7: 10	5: 10	0.647
Combined surgery				
Cataract surgery	18 (56.3)	11 (64.7)	7 (46.7)	0.621
Pupilloplasty	3 (9.4)	2 (11.8)	1 (6.7)	0.476
Ocular comorbidities	20 (62.5)	10 (58.8)	10 (66.7)	0.647
Glaucoma (n, %)	11 (34.4)	7 (41.2)	4 (26.7)	0.388
Other comorbidities	11 (34.4)	4 (23.5)	7 (46.7)	0.169
ACD (phakic state)	2.2±0.6	2.2±0.7	2.1±0.4	0.748
AXL (mm)	24.1±3.2	24.4±3.5	23.5±2.5	0.518
CCT (μm)	677±63.1	676±59.8	687±68.8	0.935
ECD (cells/mm^2^) ^a^	484±80.3	486±94.7	498±13.4	0.933
Follow-up (months)	33.3±13.7	42.3±11.8	23.0±7.1	<0.001
**Donors**				
Age (years)	56.2±4.6	57.8±4.1	54.5±4.6	0.043
Graft size (mm)	7.72±0.5	7.4±0.3	8.0±0.4	<0.001
Donor ECD (cells/mm^2^)	2812±205	2837±169	2783±242	0.469
Death-to-use time (hours)	131±11	127±12	135±8	0.051
Pre-cut-to-use time	59±15	59±15	58±15	0.951

Endo, endothelium; M, male; F, female; BCVA, best corrected visual acuity; BK, bullous keratopathy; ACD, anterior chamber depth; AXL, axial length; ECD, endothelial cell density, Other morbidities include uveitis, toxic anterior segment syndrome, epiretinal membrane, cystoid macular edema, and pseudoexfoliation syndrome etc. ECD^a^: only assessed in 8 cases (25%) owing to corneal edema

**Fig 1:**
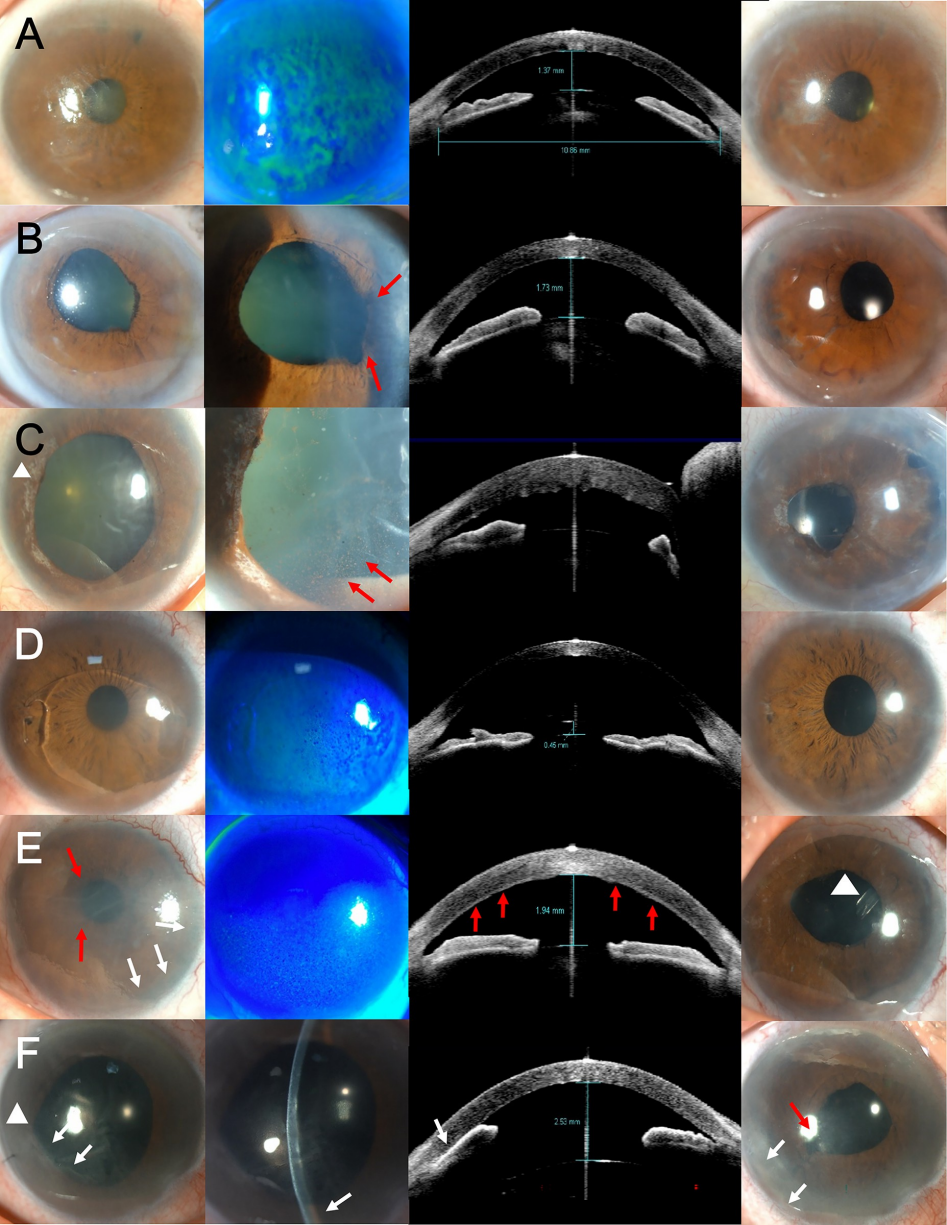
Representative photographs of patients with a concomitant ocular disease who underwent Descemet membrane endothelial keratoplasty (DMEK). First and second column: preoperative photographs of the cornea. Third column: horizontal (A–E) or vertical scans (F) of anterior segment optical coherence tomography. Fourth column: postoperative photographs of the cornea after DMEK. (A) Anterior displaced crystalline lens due to zonular weakness. (B) Posterior iris synechiae (red arrows) after acute angle-closure glaucoma. (C) Dilated and paralyzed pupil (white arrowhead), keratic precipitates (red arrows) and shallow anterior chamber due to acute angle-closure glaucoma. Pupilloplasty and cataract surgery were performed prior to DMEK. (D) Iris-fixed phakic intraocular lens (IOL)-induced iritis and bullous keratopathy. (E) Uveitis glaucoma with thickened corneal endothelium (red arrows) and peripheral anterior synechiae (white arrows). Glaucoma drainage device insertion (white arrowhead) after DMEK. (F) Toxic anterior segment syndrome and uvietic glaucoma with progressive peripheral anterior synechiae (white arrows). Dilated pupil (white arrowhead) is corrected with pupilloplasty (red arrow).

The following data were collected from the medical charts: demographic information that included the recipient’s age, sex, general medical history, ocular medical and surgical history; and ophthalmic findings that included visual acuity, intraocular pressure (IOP), endothelial cell density (ECD), central corneal thickness (CCT), ocular biometric parameters, and the follow-up period (Table 1). Concomitant ophthalmic diseases that might affect graft survival or visual acuity, which included glaucoma, uveitis, toxic anterior segment syndrome, epiretinal membranes, age-related macular degeneration, cystoid macular edema, pseudoexfoliation syndrome, pupillary abnormality, and iris synechiae are shown in Fig 1.

### Donor characteristics in overseas transport of pre-cut tissues

A total of 32 transplant grafts were supplied by the Eversight Eye Bank (Chicago, IL) using the standard procedure [26, 27]. Only donor tissues with a death-to-use period of < 7 days were imported. Double partial trephinated-grafts with S-marks were used. The size of the grafts ranged from 7.25–8.5 mm and the grafts were selected based on the recipient’s vertical and horizontal corneal dimensions, which were preoperatively measured using anterior segment optical coherence tomography (Carl Zeiss Meditec, Dublin, CA, USA). The pre-cut Descemet membranes were preserved *in situ* on top of the donor corneal stroma in Optisol (Chiron, Irvine, CA, USA). The mean pre-cut-to-use period for overseas transport was 59±15 hours. Donor tissue information, including age, graft size, donor ECD, and death-to-use period is shown in Table 1.

### Surgical interventions

Peripheral laser iridotomy or surgical iridectomy during cataract surgery was performed before DMEK. For patients with a cataract or a pupil anomaly, phacoemulsification with an intraocular lens (IOL) insertion or pupilloplasty was performed concomitantly or sequentially prior to DMEK without any adverse event. Endothelium-out grafts were delivered into the anterior chamber during DMEK using glass Jones tubes (GWSG, Hillsboro, OR) in 17 eyes from October 2016 to March 2019. After August 2019, a Coronet EndoGlide insertion device (Network Medical Products, Ripon, UK) became available in Korea; after August 2019, 15 eyes received endothelium-in grafts. The size of the recipient’s Descemet membrane stripping was usually 0.5–0.75 mm larger than that of the donor graft (8.0–9.0 mm).

**Fig 2:**
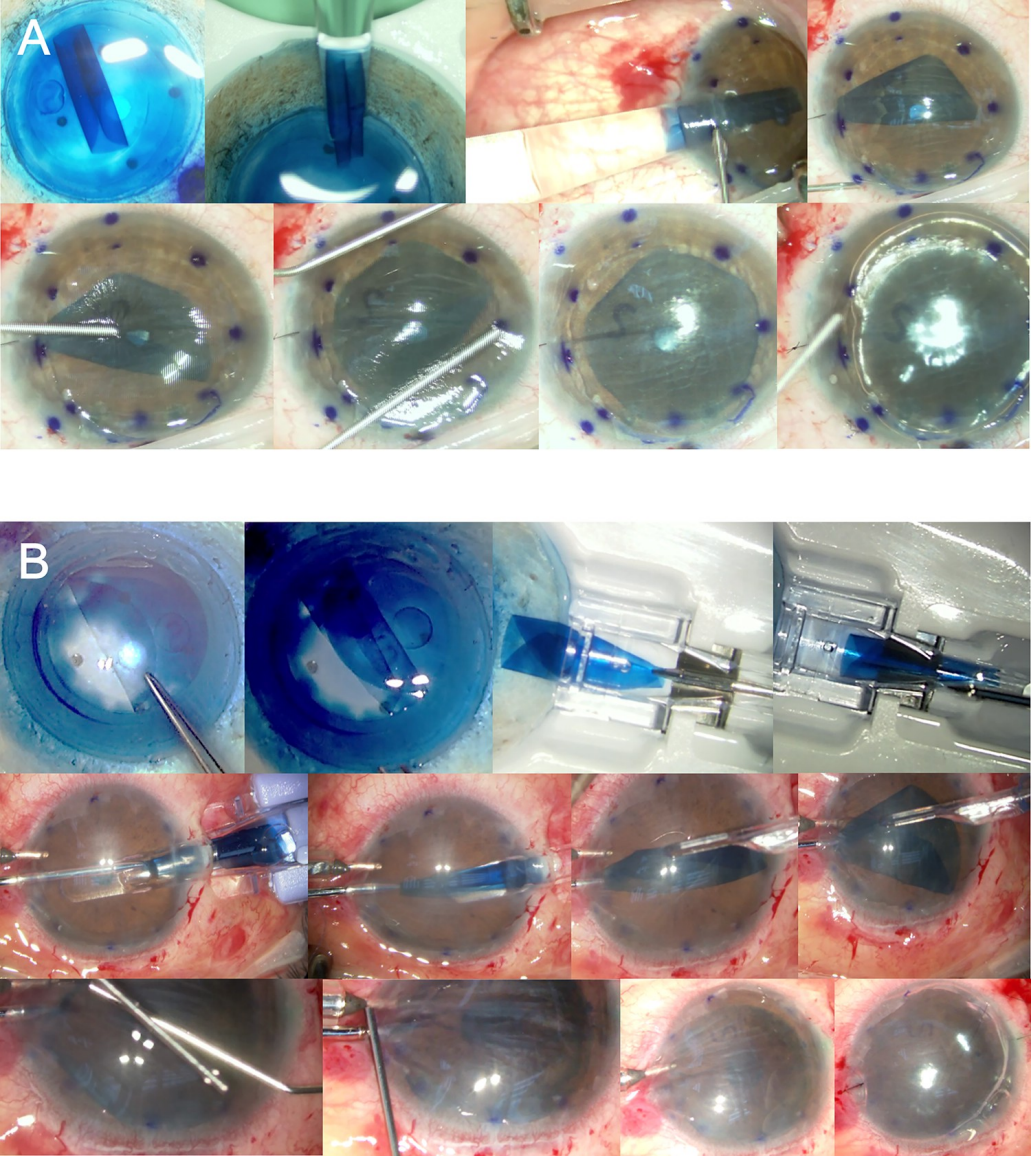
Representative photographs of surgical procedures in Descemet membrane endothelial keratoplasty (DMEK). Endothelium-out delivery with John’s tube (A) and endothelium-in technique using the DMEK EndoGlide (B).

In the endothelium-out technique, the graft was freed from the hinge and spontaneously rolled with the endothelium outward. Using a Jones tube, the graft was aspirated inside the tube with a balanced salt solution (Bausch and Lomb, Inc., Rochester, NY) and injected into the anterior chamber through a 3.5-mm corneal or scleral tunnel. The graft was unfolded using tapping procedures and was attached using an air tamponade (80–95%) after confirming that the endothelium was positioned downward by the S mark (Fig 2A) [28].

In the endothelium-in technique, the graft was harvested and tri-folded inward with its endothelium. The graft was pulled into the coronary EndoGlide, which had been filled with the balanced salt solution. The graft was delivered into the anterior chamber in the correct orientation with the endothelium facing downward through a 2.75-mm corneal or scleral tunnel. The graft was unfolded with the assistance of tapping and was attached using an air tamponade (80–95%) with a confirmation of the S mark (Fig 2B) [14]. The standard follow-up consisted of examinations at 1 day, 2 days, 1 week, 1, 3, and 6 months, and every 6 months after DMEK.

All the patients received systemic steroid treatment for more than 3 weeks. Topical 1% prednisolone acetate (Pred Forte; Allergan, Inc., Irvine, CA, USA) was administered 4 times a day and the dose was tapered down to 1/day after 12 months. Topical 0.5% moxifloxacin eye drops (Vigamox, Alcon, TX) were applied 4 times a day.

### Clinical outcome measurements

The following data were collected as the main outcomes and analyzed in each time point in a whole group and were also compared in between the endothelium‐in and endothelium‐out deliveries; graft survival, postoperative best corrected visual acuity (BCVA), ECD, CCT, re-bubbling rate and annual EC loss (ECL) rate. Early and delayed complications were additionally compared between those two groups. Visual acuities measured initially by Snellen charts were converted to logarithm of the minimum angle of resolution (logMAR) values for analysis. The proportions of BCVA ≥20/40, 20/25, or 20/20 were evaluated. Patients with amblyopia, advanced glaucoma, or macular disease that could affect visual acuity were excluded from visual acuity analysis. Increased IOP was defined as IOP > 21 mmHg. ECD was checked by specular microscopy (Konan Medical Inc., Hyogo, Japan). CCT was measured with anterior segment optical coherence tomography. The postoperative immediate and delayed complications were examined by slit-lamp microscopy (Haag-Streit AG, Koniz, Switzerland). To analyze the changes of corneal astigmatism, axial length (AXL), and mean numerical refractive errors (MNEs) in DMEK combined with cataract surgery (n = 18), an auto-kerato-refractometer (Atlas, Carl Zeiss Meditec, Dublin, CA), topography (Orbscan II Bausch & Lomb, Claremont, CA, USA), and IOL master (Carl Zeiss MeditecAG, Jena, Germany) were used. MNEs were calculated by subtracting the value of postoperatively measured refractive error from the value of the predicted refractive error calculated by IOL master (MNE = refractive errorpredicted−refractive error_postoperatively measured_) [21]. The correlation of either ΔCCT_pre-post_ (CCT_preoperative_−CCT_postoperative_) or ΔK_pre-post_ (K_preoperative_−K_postoperative_) with MNE was analyzed. In most patients, the preoperative posterior curvature (K_post_) data were not available. Therefore, postoperative K_post_ (n = 15) was compared with the K_post_ in eyes of normal controls (n = 50) in whom the parameters for cataract surgery were measured using an IOL Master 700 (Carl Zeiss Meditec AG, Jena, Germany).

Graft failure was defined as irreversible loss of corneal graft clarity despite intense medical treatment. Immunologic rejection of corneal grafts was diagnosed as the sudden onset of corneal edema developed in the presence of ocular inflammation (ciliary injection, endothelial rejection line, keratic precipitates, stromal infiltrates or cells in the anterior chamber).

### Statistical analyses

Data are presented as mean ± standard deviation where applicable. Statistical analysis was performed using SPSS Statistics 20.0 (IBM, Armonk, NY). The chi-squared test or Fisher’s exact test was used to compare the two categorical variables, and Student’s t-test was applied to compare the means of continuous variables between two groups. The Kaplan-Meier curves were used to analyze the corneal graft survival after a successful DMEK and a statistical difference in Kaplan-Meier curves was assessed by Log-rank test. Pearson’s bivariate co-relation was used between those ocular biometric parameters. A P value of < 0.05 was considered statistically significant.

## Results

### Baseline characteristics of DMEK patients

The baseline characteristics of the donors and recipients are shown in Table 1. The follow-up duration was significantly longer in the endothelium-out group compared to the endothelium-in group (42.3±11.8 months vs 23.0±7.1 months, p<0.001). There were no significant differences between those two groups in the recipients’ age, sex ratio, preoperative BCVA, anterior chamber depth, AXL, or the proportion of preoperative diagnoses with FED or BK. Combined ophthalmic diseases were present in 20 out of 32 eyes (62.5%), and the most common disease was glaucoma (n = 11, 34.4%). The other concomitant morbidities were uveitis, toxic anterior segment syndrome, iris-fixated phakic IOL-induced iritis, cystoid macular edema, epiretinal membrane, pseudoexfoliation syndrome with zonular weakness, plateau iris, and Sjögren syndrome.

Among the donors’ clinical features, the graft size was significantly larger in the endothelium-in group (8.0±0.4 mm) than in the endothelium-out group (7.4±0.3 mm, p<0.001). The mean donor death-to-use period in the endothelium-in group (135±8 hours) was similar to that in the endothelium-out group (127±12 hours, p = 0.051). The mean pre-cut-to-use period did not differ between the two groups (59±15 vs. 58±15 hours, respectively; p>0.05). There was no difference in the ECD between the two groups.

#### Overall clinical outcomes of DMEK in eyes of Asian patients

**Fig 3:**
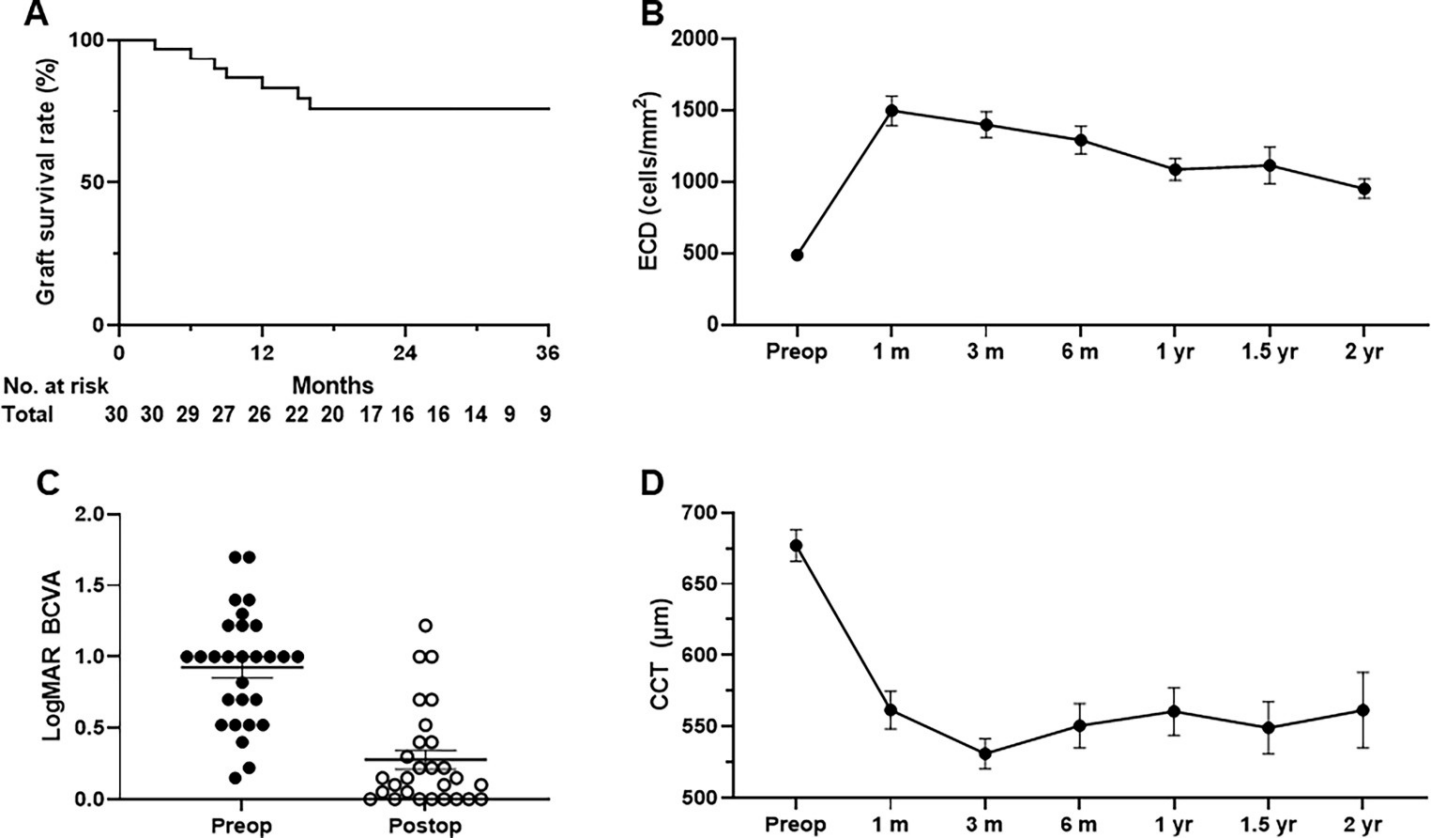
Clinical outcome of Descemet membrane endothelial keratoplasty (DMEK) using overseas transport pre-cut tissues within 3 days. Kaplan-Meier plots of overall imported graft survival rate (A), endothelial cell density (ECD) (B), best corrected visual acuity (BCVA) (C), and central corneal thickness (CCT) (D) in total cases. Data are expressed as the mean ± standard error of the mean (B, C, D).

**Fig 4:**
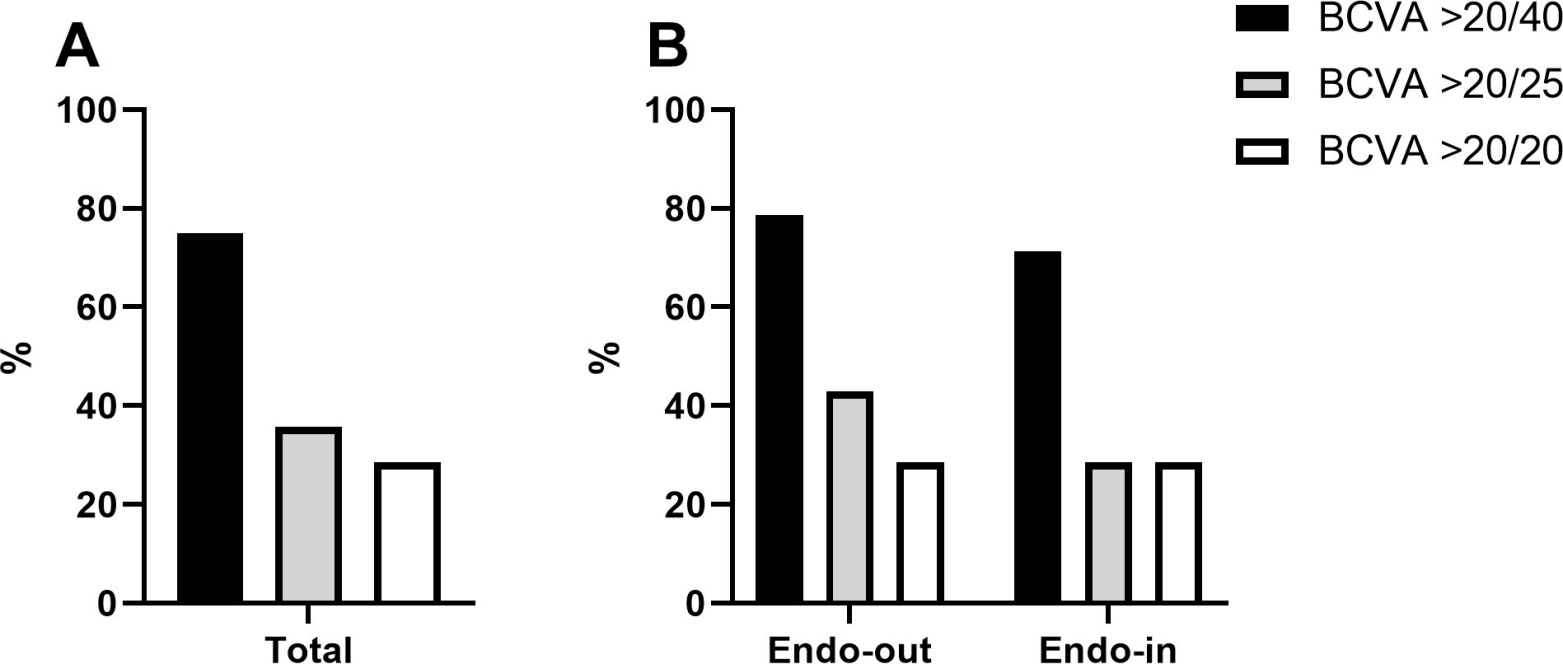
The proportion of eyes that achieved different levels of best spectacle-corrected visual acuity (BCVA) in Descemet membrane endothelial keratoplasty (DMEK) in all cases (A) and in between endothelium-out and endothelium-in groups (B). Percentages of eyes that achieved BCVA ≥20/40, ≥20/25 or ≥20/20 were not different between those two groups.

**Fig 5:**
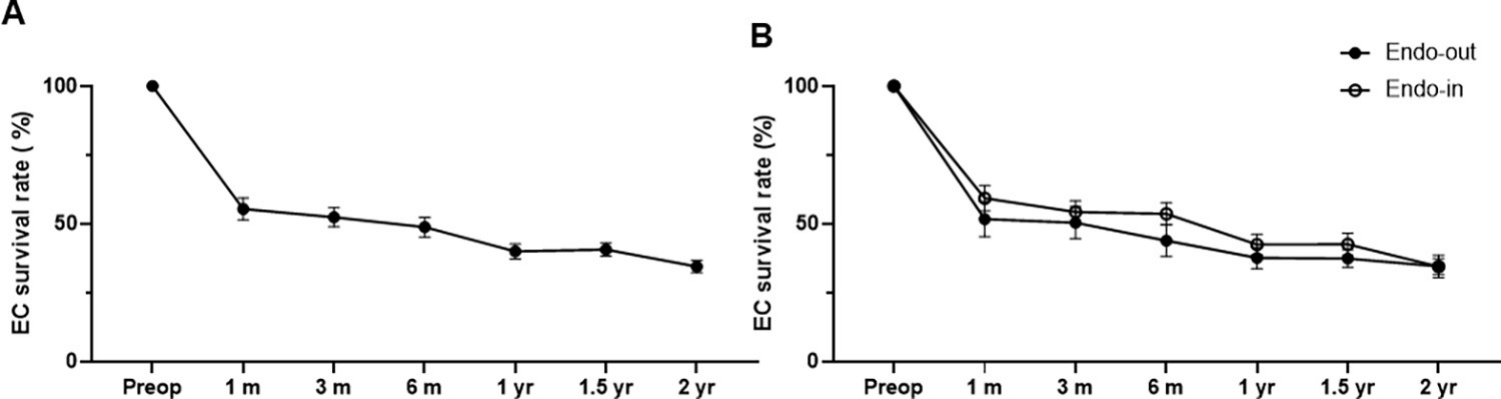
Cumulative endothelial cell survival rate of Descemet membrane endothelial keratoplasty (DMEK) using overseas transport pre-cut tissues within 3-day pre-cut-to-use period. Cumulative endothelial cell (EC) survival rate in total cases (A). Comparison of the cumulative EC survival rate after DMEK between endothelium-out (endo-out) and endothelium-in (endo-in) groups (B). Total 17 eyes (endo-out; n = 9 vs endo-in: n = 8) were included in 1.5-year FU and 14 eyes (endo-out; n = 9 vs endo-in: n = 5) were included in 2-year FU. There were no differences between those two groups over time. Data are expressed as the mean ± standard error of the mean (A, B).

The overall graft survival rate was 71.9% at the last follow-up (Fig 3A). Primary graft failure occurred in only 2 patients (6.3%); re-bubbles were performed in 11 eyes (n = 11, 34.4%). Overall graft failures during the follow-up were caused by multiple factors, which included primary graft failure (n = 2, 6.3%), uncontrolled immunological rejection (n = 1, 3.1%), persistent uveitis with progressive peripheral anterior synechia (n = 1, 3.1%), implantation of a shunt device (n = 3, 9.4%), and corneal bacterial infection (n = 2, 6.3%). Due to corneal edema, 75% of the recipients’ preoperative ECDs could not be measured (n = 24); the measurable ECD averaged 488±79 cells/mm^2^ (n = 8). After DMEK, the mean ECD was 1400±480 cells/mm^2^ at 3 months, 1291±497 cells/mm^2^ at 6 months, 1086±384 cells/mm^2^ at 1 year, and 951±252 cells/mm^2^ at 2 years (Fig 3B). The postoperative BCVA significantly increased compared to the preoperative BCVA by 89.3% (0.92±0.40 vs. 0.28±0.35, respectively; p<0.001) (Fig 3C, Table 2). Among the postoperative BCVA tests, 75% of the total eyes showed a BCVA better than 20/40, 35.7% had a BCVA better than 20/25, and 28.6% had a BCVA better than 20/20 (Fig 4A). The mean preoperative CCT (677±63 μm) decreased to 531±58 μm at 3 months, 550±88 μm at 6 months, 561±95 μm at 1 year, and 561±119μm at 2 years (Fig 3D). The annual ECL rate and number of glaucoma medications are described in Table 2. The ECL rate from the donor ECD was 47.0% at 1 month, which decreased to 28.4% by 1 year, and further decreased to 17.8% by 2 years. The cumulative EC survival rate from the ECD of the donor pre-cut tissues was shown in Fig 5A.

**Table 2:** Postoperative clinical outcomes.

	Total (n = 32)	Endo-out (n = 17)	Endo-in (n = 15)	P value
Immunologic rejection (n,%)	4 (12.5)	3 (17.6)	1 (6.7)	0.603
Primary graft failure (n,%)	2 (6.3)	2 (11.8)	0 (0)	0.486
Re-bubble (n,%)	11 (34.4)	5 (29.4)	6 (40.0)	0.529
Postoperative BCVA (logMAR)				
Improved VA (n,%)	25 (89.3)	11 (78.6)	14 (100.0)	0.222
Best BCVA during FU	0.28±0.4	0.28±0.4	0.28±0.3	0.987
No. of glaucoma drops				
Postoperative 1 yr	0.7±0.9	0.7±0.9	0.6±0.8	0.738
				
Annual ECL rate* (%)				
1m**	47.0±18	50.2±21	43.4±15	0.390
1 yr	28.4±18	29.0±20	27.7±16	0.859
2 yr	17.8±13	19.6±11	14.6±17	0.522

*ECL rate: Endothelial cell loss rate [ECL rate = (previous visit ECD-last visit ECD)x100/previous visit ECD]

**1m ECL rate [donor ECD—ECD at 1 month) x100/ donor ECD], Endo, endothelium; m, month; yr, year; BCVA, best corrected visual acuity (logMAR); FU, follow up; No, number; EC, endothelial cell; NA, not available; Total 14 eyes (endo-out; n = 9 vs endo-in: n = 5) were included in 2 year FU.

### Comparative efficacies of DMEK outcome between the endothelium-out and endothelium-in deliveries

**Fig 6:**
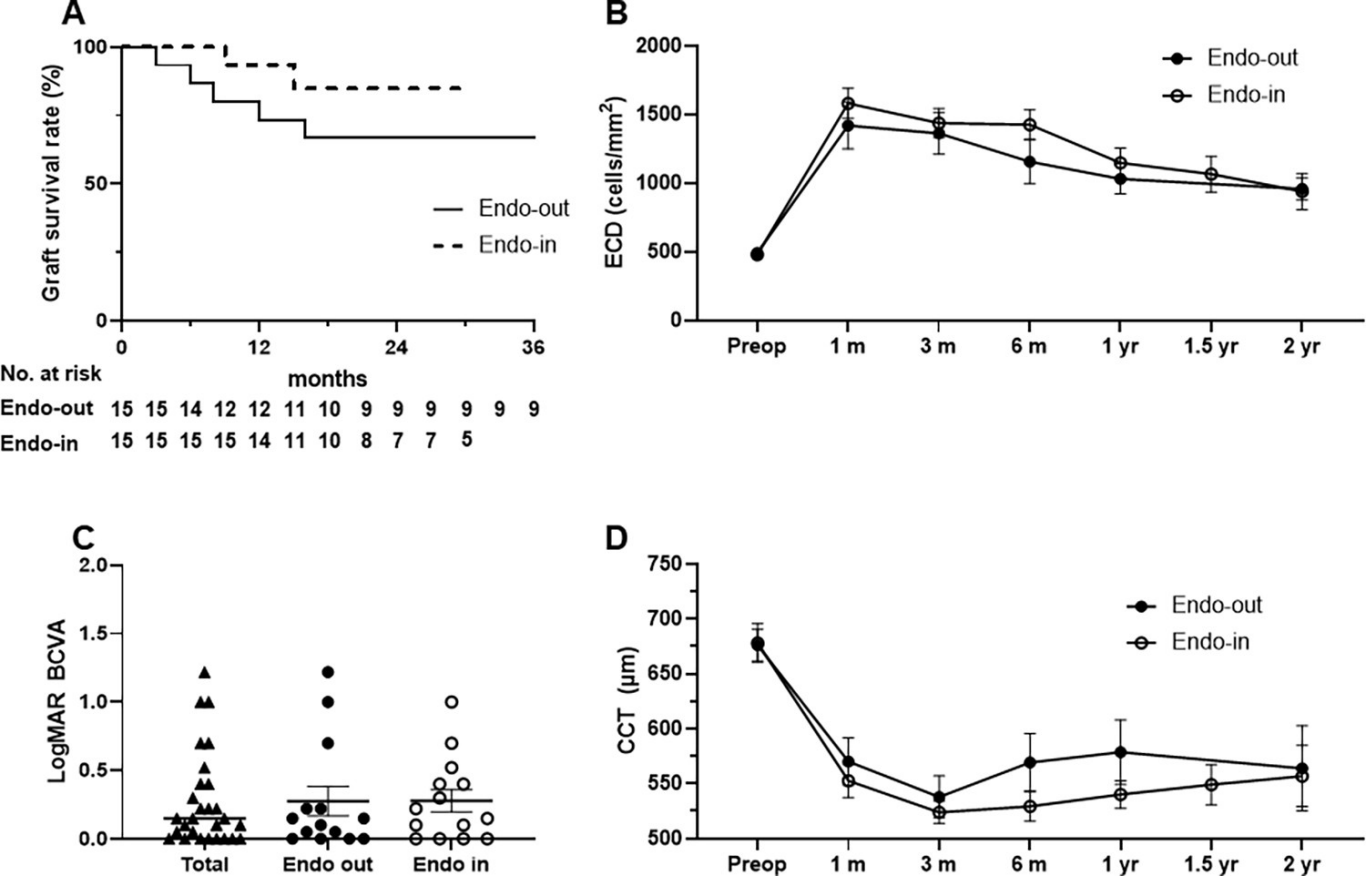
Comparison of the clinical outcome of descemet membrane endothelial keratoplasty (DMEK) between endothelium-out (endo-out) and endothelium-in (endo-in) groups. Kaplan-Meier plots of graft survival rate (A), endothelial cell density (ECD) (B), best corrected visual acuity (BCVA) (C), and central corneal thickness (CCT) (D). There were no differences between those two groups over time. Data are expressed as the mean ± standard error of the mean (B, C, D).

The overall survival rate was 66.7% in the endothelium-out group and 86.7% in the endothelium-in group (Fig 6A). The mean survival times were 32.2±19.7 and 21.0±7.9 months in the endothelium-out and endothelium-in groups, respectively, without a significant difference (p = 0.219, Fig 6A). The EC survival rate, ECD and CCT in the endothelium-in group at each time point were comparable to those in the endothelium-out group (p>0.05, Figs 5B, 6B and 6D). The postoperative BCVA was 0.28±0.4 in the endothelium-out group and 0.28±0.3 in the endothelium-in group (p = 0.987, Fig 6C). The percentage of eyes with a BCVA better than 20/40 was 78.6% in the endothelium-out group and 71.4% in the endothelium-in group (p>0.05). The proportion of eyes with BCVA better than 20/25 was 42.9% and 28.6% in the endothelium-out and endothelium-in groups, respectively (p>0.05). The fractions of eyes with BCVA better than 20/20 were 28.6% and 28.6% in the endothelium-out and endothelium-in groups, respectively (p>0.05, Fig 4B).

As shown in Table 2, the proportions of immunological rejection, primary graft failure, and re-bubble rate did not differ significantly between the endothelium-out and endothelium-in groups (p>0.05). The annual ECL rate also did not differ between the two groups (p>0.05).

### Postoperative complications after DMEK in endothelium-out and endothelium-in deliveries

The immediate and delayed postoperative complications following DMEK are presented in Table 3. A transiently increased IOP was the most common immediate postoperative adverse event (21.9%) and was controlled with anti-glaucoma medication within a day. Secondary glaucoma was the most common delayed complication (21.9%) (Table 3), and 3 patients had a glaucoma drainage device implanted for uncontrolled glaucoma. Due to intraoperative iris bleeding, one patient had anterior chamber hyphema-inducing inferior partial graft detachment in the endothelium-in group; however, spontaneous resolution was observed. Fibrin formation by intensive inflammation was observed in 2 eyes of the endothelium-out group and was successfully managed using topical and oral corticosteroids. Progressive peripheral anterior synechiae developed after DMEK in a patient with previous toxic anterior segment syndrome after cataract surgery. In the endothelium-out group, 2 patients developed corneal infectious ulcers at 6 months, which were resolved with anti-bacterial treatment. The complication rates did not differ between the endothelium-in and endothelium-out groups (p>0.05, Table 3).

**Table 3:** Postoperative complication after DMEK.

	Endo-out (n = 17)	Endo-in (n = 15)	P value
< 24hrs			
Hyphema (n, %)	0 (0)	1 (6.7)	0.469
Fibrin (n, %)	2 (11.8)	0 (0)	0.486
IIOP (n,%)	4 (23.5)	3 (20.0)	1.000
24 hrs < < 1 week			
Pupillary block* (n,%)	0 (0)	1 (6.7)	0.469
> 1 week			
Secondary glaucoma (n,%)	3 (17.6)	4 (26.7)	0.678
Progressive synechiae (n,%)	0 (0)	1 (6.7)	0.469
Partial detachment (n,%))	0 (0)	1 (6.7)	0.469
Infection (n,%)	2 (11.8)	0 (0)	0.486

DMEK, Descemet membrane endothelial keratoplasty; Endo, endothelium; hr, hour; IIOP, increased intraocular pressure; Pupillary block* was developed after re-bubbling.

### Refractive status and correlation of biometric factors with refractive errors

**Fig 7:**
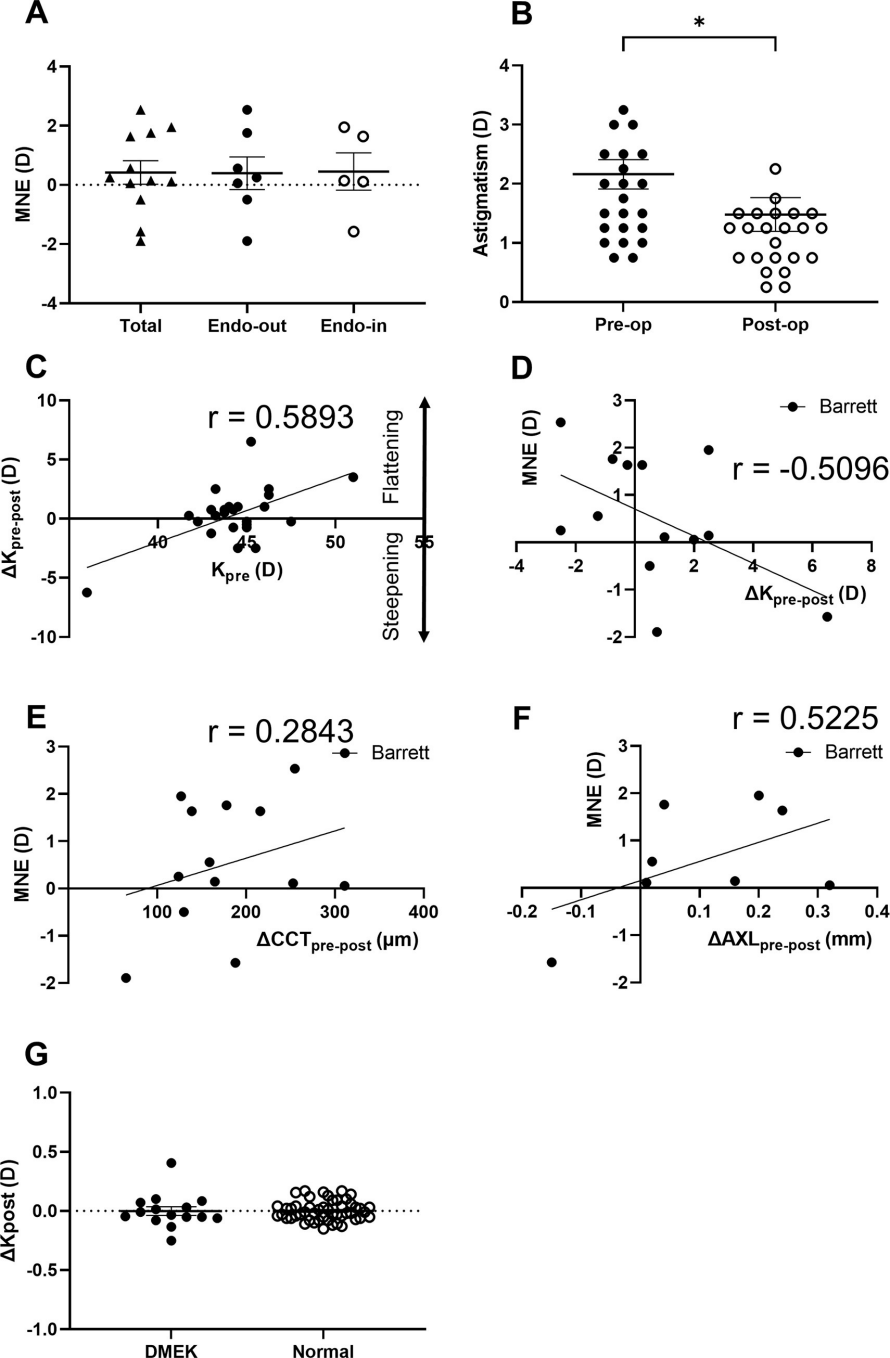
Change in the refractive parameters following Descemet Membrane Endothelial Keratoplasty (DMEK). The mean numerical refractive error (MNE) was calculated by subtracting achieved postoperative spherical equivalents from attempted predicted refraction (MNE = refraction_predicted_−refraction_postoperative measured_). mean MNEs using Barrett Universal II formula were +0.42±1.4D in total group, +0.40±1.5D in endothelium-out group and +0.45±1.4D in endothelium-in group (A). Postoperative keratometric astigmatism was significantly reduced after DMEK (p = 0.010, B). Scatterplot presents positive correlations of preoperative corneal power (K) with ΔK_pre-post_ (K_preoperative_−K_postoperative_) measured by keratometry (p = 0.002, C). Scatterplot shows MNE was not correlated with ΔK_pre-post_, ΔCCT_pre-post_ and ΔAXL (axial length _preoperative_−axial length _postoperative_) (*p*>0.05, E). Graphs compares the changes of posterior corneal curvature (ΔK_post_) in eyes with DMEK to ΔK_total-ant_ in normal eyes, which were measured by IOLMaster 700 (G). Data are expressed as the mean ± standard error of the mean (A, B, G).

At postoperative 3 months, the mean MNEs were +0.42±1.4 D in total (Barrett Universal II), and +0.40±1.5 D and +0.45±1.4 D in the endothelium-out and endothelium-in groups, respectively (Fig 7A). Postoperative keratometric astigmatism (1.3±1.6 D) was significantly reduced following DMEK (preoperative: 2.2±1.2 D, p = 0.010; Fig 7B). ΔK_**pre-post**_ was positively correlated with K_**preoperative**_ (r = 0.5893, p = 0.002; Fig 7C), suggesting that the steeper the preoperative corneal anterior curvature, the larger anterior curvature changes following DMEK. Neither ΔK_**pre-post**_ nor ΔCCT_**pre-post**_ was correlated with the MNE calculated using the Barrett Universal II formula (p>0.05; Fig 7D and 7E). MNE also did not correlate with the ΔAXL (p>0.05; Fig 7F). Mean value of the K_**post**_ in the eyes following DMEK was 0.01±0.15, and it was not different from the K_**post**_ in normal controls (+0.01±0.1D, *p* = 0.941, Fig 7G).

## Discussion

This study demonstrates that 1) the clinical efficacy and safety of DMEK in eyes of Asian patients using imported pre-cut tissues with a < 3-day pre-cut-to-use time are comparable to those in penetrating keratoplasty for BK, 2) the efficacy of the DMEK endothelium‐in method is comparable to that of the endothelium‐out method, and 3) a hyperopic shift by +0.42 D and a significant reduction of corneal astigmatism were observed. Corneal edema-induced anterior curvature changes may not affect the refractive error. However, the ECL within a month and the early annual rates of ECL were greater than those previously reported [17, 29–31].

Since endothelial keratoplasty, including DMEK, DSAEK, and ultrathin-DSAEK, results in better visual acuity and lower graft rejection than those by penetrating keratoplasty [2, 3, 7], endothelial keratoplasty is currently preferred for both BK and FED despite the challenging surgical technique. For DMEK in Western eyes, the 1- and 5-year survivals of grafts are reported to be 94% and 83% [3, 32, 33]. The central ECD of the graft decreased by 35–38% at 6 months and by 48%–59% at 5 years in DMEK for Western eyes [32, 34]. The annual loss rate of ECD in the graft following 1 year was -8% to -10% [32]. The proportion of visual acuity better than or equal to 20/40 has been reported as 88% and 96% in reports with more cases of FED [32, 33]. In addition to the DMEK procedures being more challenging, ECL may be greater in Asian eyes than in Western eyes due to their tight small fissures, shallow anterior chamber, high vitreous pressure, thicker pigmented iris, and the effortless formation of fibrin and posterior synechiae thereafter [8]. Considering the donor shortage and rarity of FED in Asia [10, 11, 35], a lack of experience due to the low surgical volume could also be related to the surgeon’s technical difficulties and be a barrier in performing DMEK.

Initial ECL at 6 months varies from 21 to 56% in reports, based on the proportion of the included FEDs and the various techniques of graft delivery to the anterior chamber [8, 9, 16, 36, 37]. In these studies, either a donor graft was manually prepared in the operating room directly prior to surgery without any intervening period [9, 16, 36, 37], or a pre-stripped donor was provided by the Eye Bank without information on the pre-cut-to-use period [8]. In the present study, the ECD at 1 month decreased by 47% from the donor ECD before the pre-cut procedure, which is a larger reduction compared to those in previous studies [15, 36]. It may be caused by the long pre-cut-to-use time (> 48 hours), the anatomical challenges in Korean eye, or the effect of the initial learning-curve. Moreover, relatively higher ECL rate and lower survival than those of previous Western studies [15, 32, 34] may have been affected by the high proportion of combined ocular morbidities (62.5%) including glaucoma (34%) and uveitis (18.8%).

Advanced FED with stromal edema was included in this study, whereas FED eyes without significant corneal edema were included in reports from Western countries [24, 25, 34]. In this study, the ECL over time was not different between the BK and grade-4 FED (FED vs BK; 44.8±20% vs 49.0±18% at 1month; 28.1±18% vs 26.9±18% at 1^st^ year; 16.5±11% vs 17.8±12% at 2^nd^ year, *p* > 0.05). A high proportion of BK inclusions (62.5%) also contributed to the high ECL at the early stage. However, similar to a DSAEK study using imported corneas [38], this study also presented a comparable survival rate with restoration of good visual acuity, suggesting that DMEK within a 3-day pre-cut-to-use period might work well in eyes of Asian patients. Since the survival rates of penetrating keratoplasty for BK were 89.8% for the first year and 47.2% for the fifth year [39], the survival of DMEK in this study showed comparable survival to that in PKP for BK.

Currently, graft delivery and unfolding procedures need to be standardized to improve the surgical outcomes. Critical steps are still performed in variable ways among different surgeons. To minimize either delivery-associated or tapping-induced ECL, we compared the surgical outcomes between the endothelium-in and endothelium-out deliveries. The visual acuity, ECL, and CCT did not differ between the two methods. Recently, *in vitro* studies discovered that ECL with the tri-folded endothelium-in method was comparable to that with the scroll-based endothelium-out method [40–42]. Moreover, recent data from the Busin group using tri-folded endothelium-in pull-through delivery demonstrated sustained survival [15]. Since the folding of the graft with the endothelium in method reduces frictional damage to the wall of the insertion cartridge, plastic cartridges, such as the IOL cartridge or EndoGlide can be used. Glass injectors, such as the Geuder cannulas or modified Jones tubes, are used for scrolled grafts with the endothelium-out method. Clinical studies have also presented acceptable ECL rates in DMEK with the endothelium-in delivery [15, 16, 43, 44]. In Korea, Geuder cannula is not commercially available. At first, we used modified Jones for a delivery of endothelium-out graft through the 3.5 mm wound. During the inserting procedure, anterior chamber tends to be easily collapsed due to larger wound, which leads to disperse iris pigments and makes unfolding procedure difficult in small-dimension eyes. After EndoGlide is commercially available in Korea, we used EndoGlide to deliver endothelium-in graft through the 2.75 mm wound and found that anterior chamber appears to be more stable with EndoGlide compared with modified Jones tube in small dimension eyes. Therefore, we aimed to compare the in vivo outcome in between endothelium-out and endothelium-in with different delivery method.This study supports the previous study by Price et al that showed no differences between the endothelium-in and endothelium-out delivery [17]

Since the dark pigmented iris blocks visibility to the graft, graft positioning with an appropriate orientation is difficult in eyes of Asian patients. Therefore, the tri-folded endothelium-in method with the assistance of graft-grabbing forceps may be an easier procedure with fewer upside-down placements than the endotheium-out method. In most cases, the scleral tunnel is preferred because of the small dimensions of the anterior chamber. Since the Geuder glass cannula is not currently available in Korea, a modified Jones tube with a 3.5 mm incision or an EndoGlide with a 2.75 mm incision are possible options. Using EndoGlide could be beneficial in maintaining anterior chamber stability during insertion compared to the Jones tube.

There were no differences in postoperative complications between the endothelium-in and endothelium-out delivery. One patient developed a pupillary block following re-bubble owing to discharge and early ambulation of the patient. Inferior iridectomy is usually recommended for preventing air- or gas-induced pupillary blocks [45–47]. However, peripheral iridectomy-less DMEK has also been reported without a pupillary block [48]. Since half the amount of injected air is absorbed within 12–24 hours [49, 50], peripheral iridectomy might not be necessary if the patient can maintain a supine position on the operative day for more than 6 hours. However, severe postoperative inflammation could cause fibrin formation when combined with uveitis [51]. Fibrin formation around the pupil or intraoperative bleeding can induce a pupillary block. Therefore, a peripheral iridectomy should be performed for early ambulation of the host or for the reduction of a pupillary block by air or fibrin.

This study demonstrated a hyperopic shift (+0.42 D) which corresponded with the refractive outcomes (+0.55 ~ +1.12 Diopter) of previous studies following triple DMEK [20, 22, 52, 53]. The causes of hyperopic shift remain controversial. Thicker corneal thickness [20], flatter posterior center curvature [23], preoperative posterior curvature [22], posterior-to-anterior corneal curvature radii ratio, and oblate posterior corneal profile [54] are reported to be involved in the hyperopic shift. In this study, although the positive correlation between K_preoperative_ and ΔK_pre-post_ indicates anterior curvature changes between before and after DMEK, changes of neither CCT nor anterior curvature correlated with the refractive shift, which supports the study by Augustine et al [22]. Another possibility is that keratometry or axial length are measured less accurately owing to epithelial edema of BK, unlike the previous studies that included patients with FED who could maintain a regular anterior surface. However, we found no difference in the AXL between pre- and post-DMEK (S1 Fig), nor the correlation of the Δ AXL with the refractive shift. There was no difference in the posterior corneal curvature (K_post_) between the post-DMEK and normal control eyes, suggesting that the graft does not induce a major change. However, we could not evaluate directly whether the individual changes of the posterior curvature could affect the refractive shift. Considering that previous studies show that the posterior cornea curvature correlates with the hyperopic shift of post-DMEK eyes [22, 23, 54], the posterior curvature may be a weighting factor in the refractive shift.

This study has several limitations. Owing to the limitations of its retrospective design, some of the data were missing. The small sample size owing to the low popularity of DMEK surgery could have affected the statistical power. However, considering that other studies comparing two groups with similar sample sizes [40, 42, 55], the sample size of this study seems to be adequate to render a statistically sufficient power. Due to rarity of FED cases in Asia, inevitably, the indications for surgery have diversified. Given that there are other studies that have been conducted with various surgical indications, so our study also worthy to compare the clinical outcomes [36, 56–58]. In addition, the follow-up durations differed between the two groups. Both limited and different follow-up durations may increase the risk of bias in the survival analysis. Finally, ΔK_post_ was not measured since the preoperative data were not available. Therefore, further studies to assess the correlation of the Δ K_post_ with MNE are warranted. Lastly, DMEK was performed by a professor (MK Kim) who had previous experience of performing approximately 1000 penetrating keratoplasties and 30 deep anterior lamellar keratoplasties. Before starting DMEK, the professor had undergone hands-on training programs by a DMEK wet lab course conducted by the Gerrit Melles of Netherlands Institute for Innovative Ocular Surgery (NIIOS) Academy in Rotterdam. This study reported an incidence of learning curve related-early graft failure of 6.3%, which was comparable to the previous incidences of learning curve related-early graft failure of 7.69 to 4% [59–62]. Although this study was limited by the early loss of ECD, which could be affected by the learning-curve effect, it is still noteworthy since it reports that imported pre-cut tissue is feasible for DMEK in Asian countries where the shortage of corneal donor limits its popularity.

In conclusion, performing DMEK using imported pre-cut tissue with a ≤ 3-day pre-cut-to-use period may be a feasible option for either BK or FED in eyes of Asian patients. Additionally, the tri-folded endothelium-in method seems to be comparatively effective to the endothelium-out method for DMEK with imported pre-cut tissue. A hyperopic shift may not be related to the anterior curvature changes.

## Supporting information

S1 Fig Graphs comparing the changes of the axial length (AXL) in eyes with Descemet membrane endothelial keratoplasty (DMEK) between preoperative (pre-op) and postoperative (post-op) groups, which were measured by IOLMaster 700. Data are expressed as mean ± standard error of the mean.
(TIF)
