# Supplementary figures and images for: Clinical outcome of Descemet membrane endothelial keratoplasty (DMEK) with imported donor corneas in eyes of Asian patients; endothelium‐in versus endothelium‐out method
Source: PLoS One. 2022 Jun 30;17(6):e0270037. doi: 10.1371/journal.pone.0270037 (PMC9246236; doi:10.1371/journal.pone.0270037)

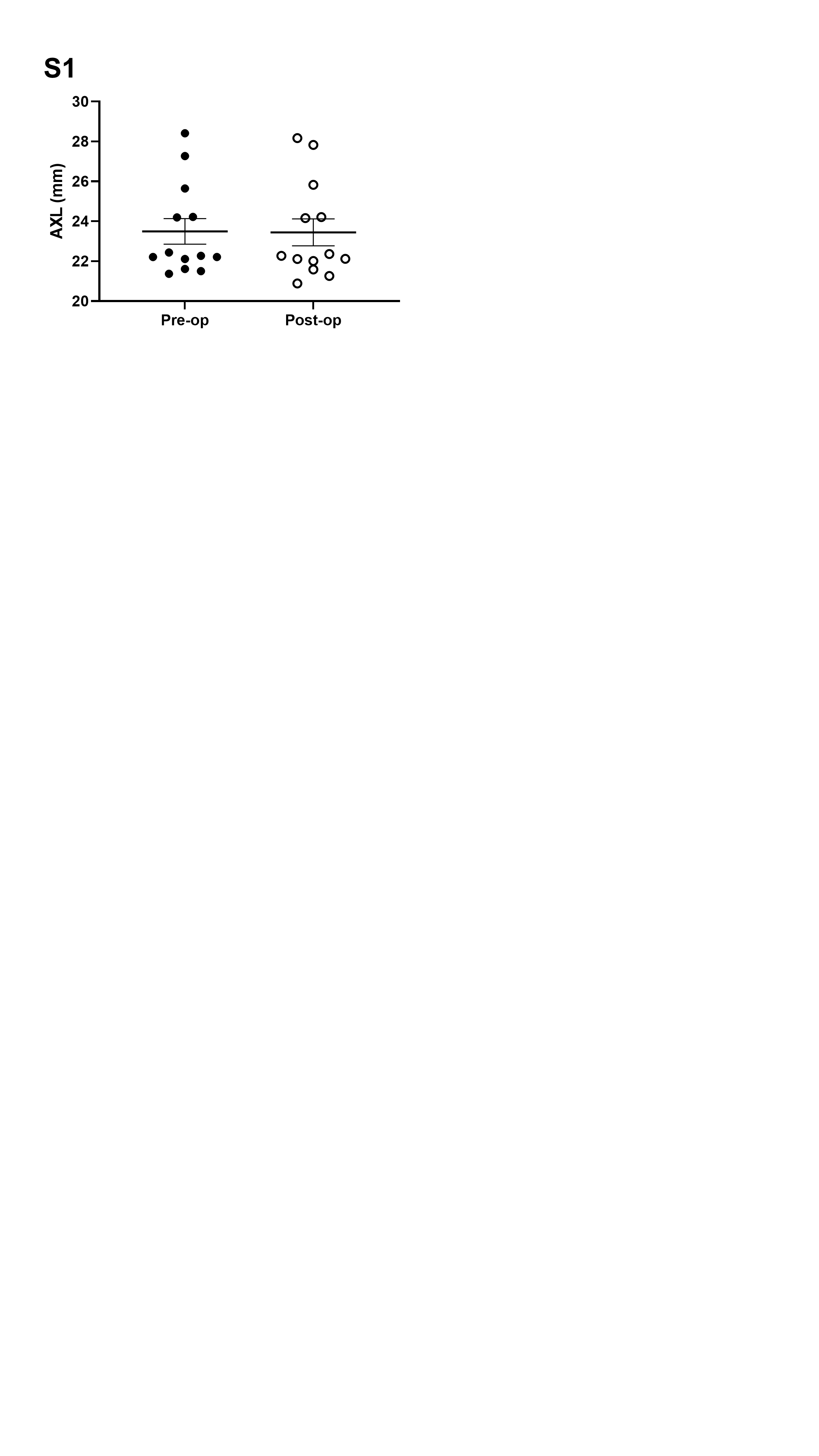

Supplement: S1 Fig — Data are expressed as mean ± standard error of the mean. (TIF) [file pone.0270037.s001.tif]
